# A Scoping Review of the Use of Pioglitazone in the Treatment of Temporo-Mandibular Joint Arthritis

**DOI:** 10.3390/ijerph192416518

**Published:** 2022-12-09

**Authors:** Natalia Turosz, Kamila Chęcińska, Maciej Chęciński, Monika Kamińska, Zuzanna Nowak, Maciej Sikora, Dariusz Chlubek

**Affiliations:** 1 Ortomania, Bartosza Głowackiego 6/1, 30-085 Kraków, Poland; 2 Department of Glass Technology and Amorphous Coatings, Faculty of Materials Science and Ceramics, AGH University of Science and Technology, Mickiewicza 30, 30-059 Kraków, Poland; 3 Department of Oral Surgery, Preventive Medicine Center, Komorowskiego 12, 30-106 Kraków, Poland; 4 Collegium Medicum, Jan Kochanowski University, aleja IX Wieków Kielc 19A, 25-317 Kielce, Poland; 5 Department of Temporomandibular Disorders, Medical University of Silesia in Katowice, Traugutta sq.2, 41-800 Zabrze, Poland; 6 Department of Maxillofacial Surgery, Hospital of the Ministry of Interior, Wojska Polskiego 51, 25-375 Kielce, Poland; 7 Department of Biochemistry and Medical Chemistry, Pomeranian Medical University, Powstańców Wielkopolskich 72, 70-111 Szczecin, Poland

**Keywords:** temporomandibular joint, arthritis, inflammation mediators, thiazolidinediones, pioglitazone

## Abstract

Thiazolidinediones (TZDs) are a group of diabetes medications currently being investigated for anti-arthritis effectiveness, one of which is pioglitazone. The purpose of this scoping review is to evaluate the potential use of pioglitazone in the treatment of temporomandibular joint (TMJ) arthritis. The criteria of eligibility were studies with the diagnosis of arthritis and pioglitazone treatment with a change in any inflammation index as an outcome. Of the 1169 records initially identified following the selection process, two animal studies and four clinical studies were included in the review. Improvements from the baseline were observed in each treatment group for each inflammation indicator. The results of the animal studies on the temporomandibular joints and on patients with rheumatoid and psoriatic arthritis indicate that the drug in question may have potential to treat arthritis, including within the temporomandibular joint.

## 1. Introduction

### 1.1. Rationale

Thiazolidinediones (TZDs, glitazones) are five-membered carbon-ring molecules, which are oral antihyperglycemic agents [1,2,3]. These drugs have been used in the medicating of diabetes mellitus since the 1990s [4]. The TZDs currently registered for diabetes treatment include pioglitazone and rosiglitazone [5]. Despite initial concerns about its carcinogenicity, pioglitazone is considered a safe drug based on many studies [6,7,8,9].

The action of TZDs is based on the activation of peroxisome proliferator-activated receptors (PPARs), which reduce insulin resistance, modify adipocyte differentiation, inhibit VEGF-induced angiogenesis, decrease leptin levels, reduce levels of various interleukins, and increase adiponectin levels [10,11,12,13,14]. The side effects of TZD intake include water retention and reduction in bone mineral density resulting in increased fracture risk, particularly in women, which may overlap with natural postmenopausal endocrine changes [15,16,17]. The only approved use of TZDs is currently to treat type 2 diabetes mellitus [1,2,3]. Albeit, experimental studies have been carried out on the therapeutic effect of TZDs in the treatment of numerous other diseases, such as polycystic ovary syndrome, ovarian hyperstimulation syndrome, nonalcoholic steatohepatitis, autism, psoriasis, and arthritis [18,19,20,21,22].

Arthritis is a collective term for diseases that manifest as inflammation that affects the joints [23,24]. The most common types of arthritis are osteoarthritis, rheumatoid arthritis, juvenile idiopathic arthritis, septic arthritis, ankylosing spondylitis, Still’s disease, psoriatic arthritis, gout, and pseudogout [25,26,27]. The local symptoms of arthritis are articular pain, joint stiffness, swelling, and dysfunction which may be complemented by systemic symptoms, in particular fatigue and weight loss [28,29]. Its diagnostics are based on clinical examination, blood tests, and imaging examinations, including classical and three-dimensional radiology and ultrasound [30]. Treatment methods that may be used include physical therapy, exercise, diet, oral and topical medications, and surgery, including arthroplasty [31,32]. Pharmacological treatments mainly involve acetaminophen, non-steroidal anti-inflammatory drugs, corticosteroids, monoclonal antibodies, and disease-modifying antirheumatic drugs [33]. Despite various medications and protocols for their administration, ongoing trials continue to investigate other innovative drugs, which may be more effective and have fewer side effects [34,35,36,37,38].

One of the goals of osteoarthritis pharmacotherapy is to reduce the concentration of inflammatory mediators [39,40,41,42,43,44,45,46]. Increased concentrations of inflammatory cytokines, chemokines, and growth factors accumulating in the articular cartilage matrix, and an increased duration of the inflammatory process associated with elevated levels of these factors, are responsible for joint destruction [47,48]. At the preliminary search stage, cell line studies and animal studies concerning induced systemic arthritis which each showed the reduction of inflammatory markers due to TZD intake [49,50,51,52,53,54,55,56,57,58,59,60,61,62,63,64] were identified. A pilot meta-analysis of the animal studies concerning one of the key proinflammatory mediators, tumor necrosis factor alpha (TNF-alpha) in induced systemic arthritis, presented promising results [50,53,56,57,60,62,65,66]. Higher doses of TZDs resulted in a stronger reduction of TNF-alpha concentrations, and these concentrations clearly showed a more significant decrease in joints than in blood serum [50,53,56,57,60,62,65,66,67].

Due to the proper function of healthy temporomandibular joints (TMJs), it is possible to move the mandible in all planes [68,69,70,71,72]. The main reason for the physical limitation of this mobility is the abnormalities in the structure of the bones and cartilage forming the temporomandibular joints as a result of inflammatory processes [68,72,73]. TMJ arthritis is an inflammatory disease that manifests itself as spontaneous pain in the TMJ and/or painful movement of the mandible, and it can occur with comorbidities such as rheumatoid arthritis and juvenile idiopathic arthritis [71,74,75,76,77]. Its diagnostics are based, first of all, on clinical examination and TMJ imaging [68,72]. Treatment methods for temporomandibular arthritis include, but are not limited to, pharmacotherapy, physiotherapy, splint therapy, intra-muscular injections, and intra-articular injections [69,70,71,72,78,79,80]. Despite many treatment protocols for TMJ arthritis, there is still no gold standard for its treatment [72,81,82,83,84]. Therefore, it is reasonable to continue research to develop new therapeutic approaches. In particular, the search among existing medications appears to be justified, with well-known positive and adverse effects [85]. Many authors have discussed the influence of TZDs on inflammatory mediators and the use of these drugs in the treatment of inflammatory diseases, including arthritis [86,87,88,89]. However, the potential use of pioglitazone in the treatment of temporomandibular arthritis has not yet been extensively discussed. Many years of use in another indication, the convenient oral form, and the promising results of the preliminary search encourage a summary of the current knowledge about the possible use of pioglitazone in the treatment of TMJs.

### 1.2. Objectives

The purpose of this scoping review is to identify, compare, and discuss studies that are relevant to considering the potential utility of pioglitazone in the treatment of TMJs arthritis. This review is intended to assist clinicians in planning and carrying out future research on the possibility of supplementing known TMJ arthritis therapies with oral pioglitazone intake, especially in diabetics.

## 2. Materials and Methods

The protocol of this review (originally planned as a systematic review) was registered in PROSPERO (Centre for Reviews and Dissemination, University of York, York, UK) under the number: CRD42022352664. The review was carried out following the PRISMA and PRISMA-ScR guidelines [90,91].

### 2.1. Eligibility Criteria

Inclusion and exclusion criteria have been developed following the PICOTS methodology [92]. No clinical trials have been identified for temporomandibular arthritis. It was decided to establish two similar sets of eligibility criteria and carry out two parallel scoping reviews for animal studies (Problem A) and clinical studies (Problem B). Due to the identical wording of the Intervention, Comparison, Outcomes, Timeframe and Settings criteria, they are discussed and presented in Table 1 together.

Studies were selected in which animals received pioglitazone as arthritis treatment (Problem A). In the identified studies, arthritis was pharmacologically induced in each case, as highlighted in Table 1 for clarity. Clinical trials for the treatment of any other type of arthritis in patients of any age and gender were included as Problem B. Due to the unknown mechanism of action of TZDs on the joints and the attempt to determine whether the therapy is causal or symptomatic, it was not decided to limit it to specific disease entities. The clinical diagnosis of arthritis was required to be radiologically confirmed or coexist with psoriasis.

For both Problems, to be able to compare the effectiveness of treatments in differently designed studies, the initial and final values of indicators of the severity of tissue inflammation were required to be available in the content of the report. All kinds of control groups and studies without a control group were allowed. The time frame for the included publications was not limited, but they were required to be available in English. Only research with published results was accepted, regardless of study design.

### 2.2. Information Sources and Search

A systematic search of medical databases was carried out based on 11 search engines: ACM Digital, BASE, EbscoHost, Embase, Ovid, ProQuest, PubMed, Scopus, Virtual Health Library, Web of Science, and Wiley Online Library [93,94,95,96,97,98,99,100,101,102,103]. One engine planned to be used, ScienceDirect, was omitted due to the lack of support for a query of the required complexity [104]. All final searches were made on 11 April 2022, under the same strategy:

(arthritis OR osteoarthritis OR polyarthritis OR rheumatism OR rheumatic OR rheumatoid OR gout) AND (thiazolidinedione OR thiazolidinediones OR TZD OR glitazone OR glitazones OR pioglitazone OR actos OR rosiglitazone OR avandia OR lobeglitazone OR duvie OR ciglitazone OR darglitazone OR englitazone OR netoglitazone OR rivoglitazone OR troglitazone OR rezulin OR balaglitazone OR drf-2593 OR as-605240).

The list of individual search engines and queries tailored to the specificity of these tools is presented in Table A1.

### 2.3. Selection of Sources of Evidence, Data Charting Process, and Critical Appraisal of Individual Sources of Evidence

The identified records were processed by two authors (K.C. and M.C.) using the Rayyan tool (Qatar Computing Research Institute, Doha, Qatar and Rayyan Systems, Cambridge, MA, USA). After manual deduplication, the screening was carried out, and the convergence of judges’ assessments at this stage was expressed by Cohen’s kappa coefficient. In case of discrepancy in decisions, a given record was qualified for full-text evaluation on a par with those unanimously included. Further analysis of the full texts led to the final decision on each of the reports (K.C and M.C). Data were extracted from the content of qualified publications without the use of automation tools independently by two authors (N.T. and M.C.). The risk of bias was assessed for the included studies according to the RoB2 and ROBINS-I questionnaires for randomized and non-randomized trials, respectively.

### 2.4. Data Items

The following elements characterizing reports and study groups have been extracted: (1) First author; (2) Number of patients; (3) Diagnosis; (4) Daily dose, mg; (5) Treatment duration; (6) Inflammation indicator; (7) Initial value (100%) of this indicator; (8) Final value of this indicator; (9) Initial indicator value (100%) for control group; (10) Final indicator value for control group. If the exact number of days is not specified, it is assumed that one month consists of four weeks. A visual analog scale (VAS) with a different numerical range was converted proportionally to values from 0 to 10. The C-reactive protein concentrations were converted to mg/L.

### 2.5. Statistical Analysis (Synthesis of Results)

The final value of each indicator as a percentage of its original value was used as a measure of effect. It was calculated according to the formula developed for this synthesis:

e = f/i × 100%,

where e is the effectiveness of the therapy, f is the final value of the indicator for which the effect is measured, and i is the initial value of this indicator. The variables mentioned above were fully synthesized in the table. The values of the test probability *p* < 0.05 were considered statistically significant. The selected data are discussed in the text and presented in graphic form. Statistical analysis and visualization were performed using Microsoft Office software (Microsoft, Redmond, WA, USA).

## 3. Results

### 3.1. Selection of Sources of Evidence

**Figure 1:**
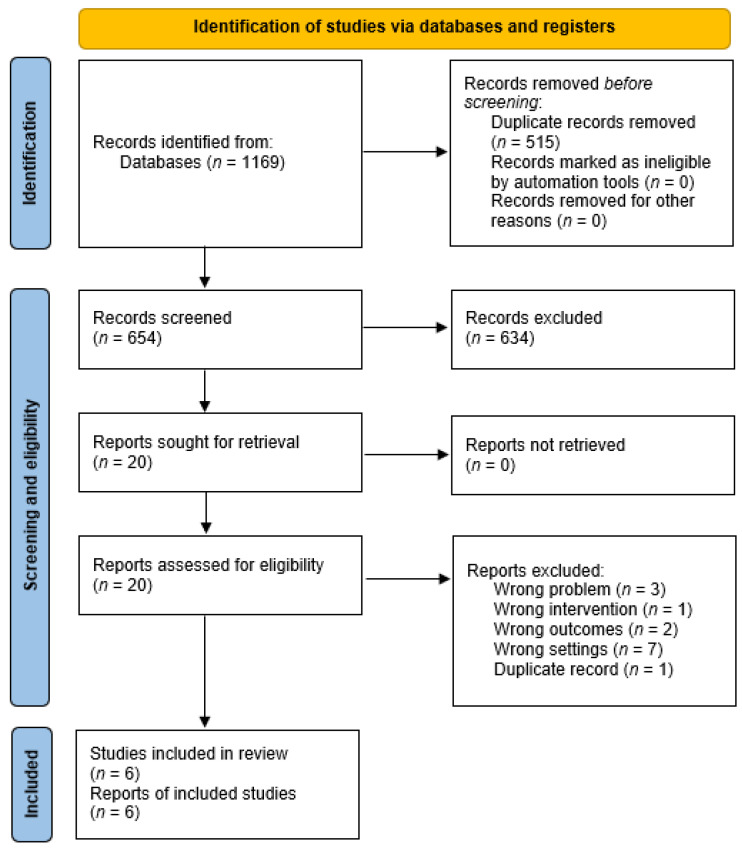
PRISMA flow diagram.

Searching medical databases using the above-mentioned search engines gave a total of 1169 records. Of these, the automation tool identified 683 potentially duplicate entries, of which 515 were manually deleted because of confirmation of duplication, leaving 168. After the deduplication process was completed, 654 records were screened. The convergence of the evaluations of two jurors at the screening stage was 99.54% (Cohen’s κ = 0.91). In total, 375 abstracts were rejected due to inadequacy problems, including diagnoses other than the predetermined criteria, and cell line studies. Due to the wrong interventions and outcomes, 11 and five reports, respectively, were excluded. The wrong type of publication was found in 265 cases, including review articles and case reports. A foreign language resulted in the abandonment of the full-text evaluation of 27 items. Twenty reports were qualified for full-text evaluation, and six of them were finally included in the synthesis. The entire selection process is shown in Figure 1. The detailed numbers of records identified by each search engine are given in Table A2. The records rejected during full-text evaluation are summarized in Table A3.

### 3.2. Characteristics of Sources of Evidence, Critical Appraisal within Sources of Evidence and Results of Individual Sources of Evidence

In total, 27 tests on various indicators of inflammation were identified in six reports. The results obtained for six study groups and six control groups are presented below (Table 2).

#### 3.2.1. Animal Studies

Two of the reports discussed the effect of pioglitazone therapy in reducing the severity of TMJ arthritis in rabbits and mice [49,50]. The study by Shiojiri et al. did not specify the number of animals in the study group [50]. In both reports, due to the specificity of the inflammation indicators, the initial values were not known, and the percentage evaluation of the effectiveness of the treatment was calculated on the basis of the final values in the study and control groups [49,50]. The number of cartilage cell layers in the study by Kałużyński et al. is the only indicator for which values greater than 100% mean therapeutic success [49]. For the remaining papers (including clinical trials), the lower the percentage value is in the last column of the table (Final value as a%), the better the treatment effect was.

#### 3.2.2. Clinical Trials

Another four reports concerned the treatment of a total of 172 patients suffering from systemic arthritis. The reports of both Shahin et al. and Bongartz et al. were ranked with a moderate risk of bias. This was due to their allocation of patients to treatment groups based on the severity of their baseline condition in the first study and with a lack of any blinding in the latter. Papers by Ormseth et al. and Marder at al. were judged to raise some concerns, but the main cause of this verdict was their missing data, despite the proper study design. In the study by Shahin et al., rheumatoid arthritis coexisted with diabetes mellitus; in Bongartz et al.’s study, patients were diagnosed with psoriatic arthritis; and in the other two clinical studies, rheumatoid arthritis was treated [65,67,105,106]. Differences in the daily doses and the number of days of treatment resulted in a difference in the total drug dose. The latter ranged from 2520 to 5040 mg [65,67,105,106]. Comparison of the baseline and end values of the arthritis indicators in the control groups using the Student’s two-sided paired T-test showed that they were not statistically significant (*p* = 0.553). The same test showed a statistical significance between the initial and final values of the severity of inflammatory mediators (*p* = 0.007). The mean improvement for all tests in all study groups was 31%.

### 3.3. Synthesis of Results of Clinical Trials

**Figure 2:**
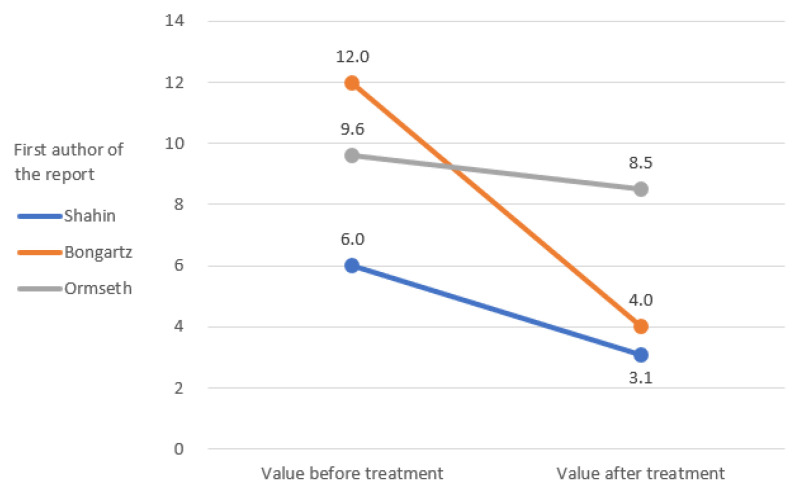
Initial and final values of tender joint count (TJC).

**Figure 3:**
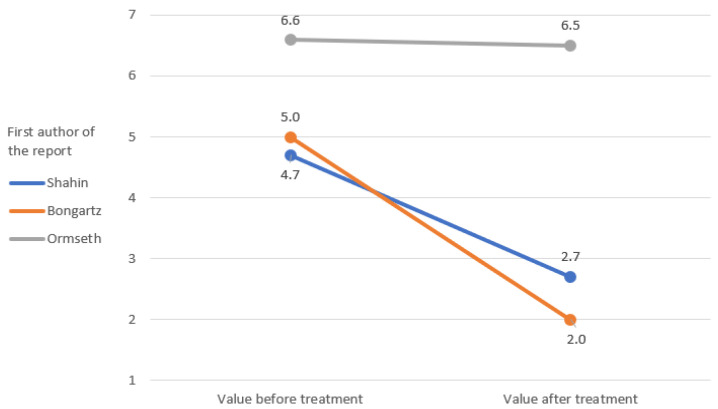
Initial and final values of swollen joint count (SJC).

**Figure 4:**
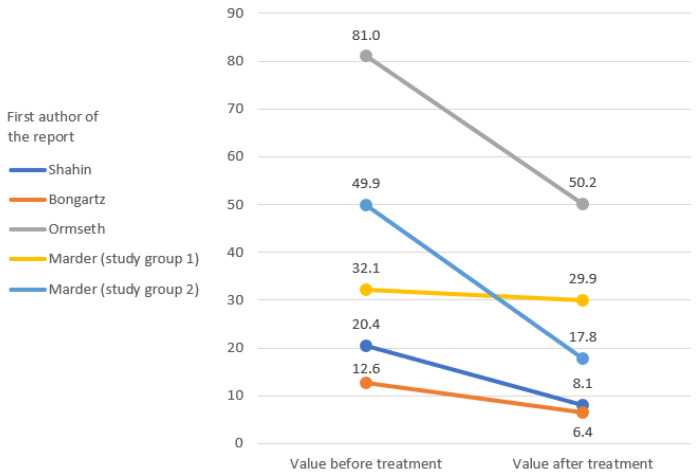
Initial and final values of C-reactive protein (CRP) [mg/L].

**Figure 5:**
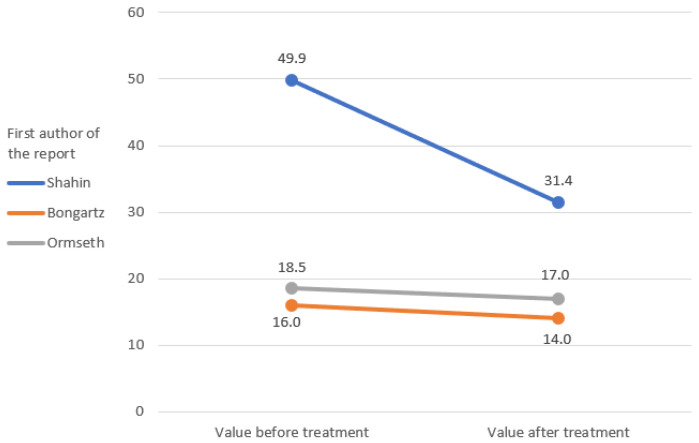
Initial and final values of erythrocyte sedimentation rate (ESR) [mm/h].

**Figure 6:**
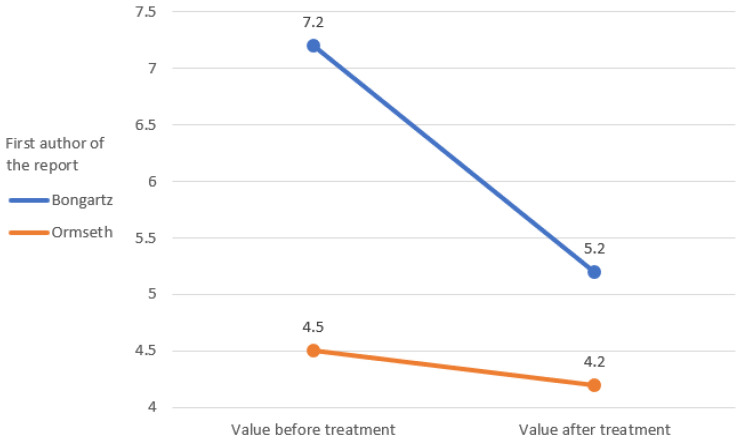
The initial and final value of the visual analog scale (VAS) pain.

The IL-6 and TNF-alpha rates were only measured in the study by Ormseth et al. [67]. The remaining markers of inflammation were repeated in the clinical reports that were analyzed, which allowed a graphical representation of the change in their rates (Figure 2, Figure 3, Figure 4, Figure 5 and Figure 6) [65,67,105,106].

## 4. Discussion

### 4.1. Summary of Evidence

At the preliminary search and selection stages of this review, cell line studies and animal studies concerning induced systemic arthritis which each showed the reduction of inflammatory markers due to TZD application were identified [49,50,51,52,53,54,55,56,57,58,59,60,61,62,63,64]. A decrease in following indicators concentrations was observed: IL-1, IL-6, interleukin-17 (IL-17), TNF-alpha, matrix metalloproteinase-1 (MMP1), matrix metalloproteinase-13 (MMP13), CRP, and ESR [49,50,51,52,53,54,55,56,57,58,59,60,61,62,63,64]. The decrease of TNF-alpha level was obtained in the study by Wang et al., who additionally noted the reduction of IL-1 [57]. Another decrease in IL-1 occurred in the study by Liu et al. [51]. A relatively small reduction of this indicator was observed in the study by Kobayashi et al., but the level of MMP-13 dropped significantly [64]. Similar results regarding this matrix metalloproteinase were obtained in the study of Zhang et al. [54]. Good effectiveness in preventing cartilage degeneration was observed in the studies by Li et al. and Kałużyński et al. [49,53]. These results confirm that TZD therapy, and particularly pioglitazone, can be beneficial in reducing commonly known indicators of arthritis [49,50,51,52,53,54,55,56,57,58,59,60,61,62,63,64]. Two of the above-mentioned studies were included in this scoping review as having TMJ treatment outcomes [49,50]. Administration of pioglitazone in the test groups gave results better than those in the control groups by about 20% to 70%, depending on the methodology [49,50]. The rosiglitazone score is approximately 55% of an improvement over a placebo [50].

The results of clinical studies show a decrease in arthritis markers with pioglitazone therapy [65,67,105,106]. No TZDs other than pioglitazone were used in the included studies [65,67,105,106]. The change of TJC and SJC values over the course of the treatment in Shahin et al.’s and Bongartz et al.’s studies is promising [65,105]. The effect in these domains was much less pronounced than in the study by Ormseth et al., although the diagnosis and total dose were consistent with those of Shahin et al. [65,67]. In both studies, patients used pioglitazone as an add-on to their baseline therapy [65,67]. There was a clear difference in the length of therapy, with the study by Shahin et al. lasting 12 weeks and Ormseth et al.’s study lasting 8 weeks [65,67]. Therefore, it should be carefully assumed that a shorter administration time, despite the higher single doses, may give a worse anti-inflammatory effect [65,67]. Similar relationships were observed for the CRP index [65,67,105]. The results of the Shahin et al. and Ormseth et al. studies again proved to be in favor of the test group in the first report [65,67]. CRP was the only common indicator for all four studies [65,67,105,106]. The weakest improvement in the CRP domain was reported in the article by Marder et al., which could not be explained by the test conditions [106]. Studies of ESR and VAS pain ratios are difficult to interpret due to the small amount of inconsistent data [65,67,105].

### 4.2. Limitations

The limitations of the evidence in this scoping review are the heterogeneity of the studies and their concentration on pioglitazone, and the almost complete omission of rosiglitazone. Moreover, no clinical trials of TMJ arthritis have been identified, and only two animal-based studies are known.

### 4.3. Conclusions

Improvements from the baseline were observed in each treatment group for each inflammation indicator due to pioglitazone therapy. In relation to the control groups, better results were achieved in all combinations, except for SJC, TNF-alpha level, and DAS28-ESR, in the study of Ormseth et al. The results of animal studies on the temporomandibular joints and on patients with rheumatoid and psoriatic arthritis indicate that the drug in question may have potential to treat arthritis, including within the temporomandibular joint. Therefore, the development and conduct of clinical trials in collaboration with rheumatologists and maxillofacial surgeons in patients receiving pioglitazone as a treatment for hyperglycemia may be a promising direction for further research. Replacing treatments such as splint therapy and intra-articular injections with oral drugs also seems theoretically possible in the future, including for non-diabetic patients, but certainly cannot be considered in the current state of knowledge.

## Figures and Tables

**Table 1:** Eligibility criteria.

	Inclusion	Exclusion
Problem A	Diagnosis of induced temporomandibular joint arthritis in animal studies	Clinical trials, cell studies
Problem B	Diagnosis of arthritis in clinical trials	Animal studies, cell studies
Intervention	Oral pioglitazone administration	-
Comparison	Any or none	-
Outcomes	Efficacy of therapy in changing the value of inflammation markers	No comparison nor initial and final values of inflammation markers
Timeframe	Any	-
Settings	Any type of primary study with published results	Language other than English; case reports and case series

**Table 2:** Study characteristics and results of individual studies.

First Author	Study Group	Diagnosis	Daily Dose, mg	Duration, Weeks	Inflammation Indicator	Control Group			Study Group		
						Initial Value (100%)	Final Value	Final Value as a%	Initial Value (100%)	Final Value	Final Value as a%
Animal studies											
Shiojiri [50]	Adult mice	Induced TMJ arthritis	30 per kg of body weight	1.5	Nitrotyrosine [arbitrary unit]	N/S	1100 *	-	N/S	900 *	81.8% compared to the control
Kałużyński [49]	10 californian white rabbits	Induced TMJ arthritis	2 per kg of body weight	4	Cartilage cell layers—transitional zone	N/S	9	-	N/S	11	122.2% compared to the control
					Cartilage cell layers—deep zone	N/S	10	-	N/S	17	170.0% compared to the control
Clinical trials											
Shahin [65]	28 patients	Rheumatoid arthritis	30	12	TJC	5.6	3.6	64.3%	6.0	3.1	51.7%
					SJC	4.1	3.1	75.6%	4.7	2.7	57.4%
					CRP [mg/L]	18.7	13.6	72.7%	20.4	8.1	39.7%
					ESR [mm/h]	32.1	21.7	67.6%	49.9	31.4	62.9%
					DAS28	4.6	4.2	91.3%	5.2	3.8	73.1%
Bongartz [105]	10 patients	Psoriatic arthritis	60	12	TJC	N/S	N/S	N/S	12.0	4.0	33.3%
					SJC	N/S	N/S	N/S	5.0	2.0	40.0%
					CRP [mg/L]	N/S	N/S	N/S	12.6	6.4	50.8%
					ESR [mm/h]	N/S	N/S	N/S	16.0	14.0	87.5%
					VAS	N/S	N/S	N/S	7.2	5.2	72.2%
Ormseth [67]	26 patients	Rheumatoid arthritis	45	8	TJC	11.5	10.4	90.4%	9.6	8.5	88.5%
					SJC	8.2	7	85.4%	6.6	6.5	98.5%
					CRP [mg/L]	77.0	82.5	107.1%	81.0	50.2	62.0%
					ESR [mm/h]	19.5	18.9	96.9%	18.5	17.0	91.9%
					VAS	4.8	4.9	102.1%	4.5	4.2	93.3%
					IL-6	8.7	6.5	74.7%	5.4	2.4	44.4%
					TNF-alpha	13.4	9.7	72.4%	9.9	9.5	96.0%
					DAS28-CRP	4.6	4.5	97.8%	4.4	4.0	90.9%
					DAS28-ESR	4.9	4.6	93.9%	4.6	4.4	95.7%
Marder [106]	108 patients	Rheumatoid arthritis	45	13 *	CRP [mg/L] (study group 1)	56.7 *	73.2 *	129.1%	32.1 *	29.9 *	93.1%
					CRP [mg/L] (study group 2)	45.5 *	37.8 *	83.1%	49.9 *	17.8 *	35.7%
					DAS28 (study group 1)	3.6 *	3.3 *	91.7%	3.2 *	2.9 *	90.6%
					DAS28 (study group 2)	3.4 *	3.3 *	97.1%	3.3 *	2.8 *	84.8%

TMJ—temporomandibular joint; TJC—tender joint count; SJC—swollen joint count; CRP—C-reactive protein level; ESR—erythrocyte sedimentation rate; DAS28—disease activity score for 28 joints; VAS—patient’s assessment of pain using visual analog scale; IL-6—interleukin 6 level; TNF-alpha—tumor necrosis factor-alpha level; *—approximate value; N/S—not specified.

## Data Availability

All the collected data and the results of their processing are presented in this article.

## Appendix A. For Materials and Methods

**Table A1:** Search engines and search strategies.

Search Engine	Search Strategy
ACM Digital	[[All: arthritis] OR [All: osteoarthritis] OR [All: polyarthritis] OR [All: rheumatism] OR [All: rheumatic] OR [All: rheumatoid] OR [All: gout]] AND [[All: thiazolidinedione] OR [All: thiazolidinediones] OR [All: tzd] OR [All: glitazone] OR [All: glitazones] OR [All: pioglitazone] OR [All: actos] OR [All: rosiglitazone] OR [All: avandia] OR [All: lobeglitazone] OR [All: duvie] OR [All: ciglitazone] OR [All: darglitazone] OR [All: englitazone] OR [All: netoglitazone] OR [All: rivoglitazone] OR [All: troglitazone] OR [All: rezulin] OR [All: balaglitazone] OR [All: drf-2593] OR [All: as-605240]]
BASE	tit:(arthritis OR osteoarthritis OR polyarthritis OR rheumatism OR rheumatic OR rheumatoid OR gout) AND (thiazolidinedione OR thiazolidinediones OR TZD OR glitazone OR glitazones OR pioglitazone OR actos OR rosiglitazone OR avandia OR lobeglitazone OR duvie OR ciglitazone OR darglitazone OR englitazone OR netoglitazone OR rivoglitazone OR troglitazone OR rezulin OR balaglitazone OR drf-2593 OR as-605240)
EbscoHost	TI (arthritis OR osteoarthritis OR polyarthritis OR rheumatism OR rheumatic OR rheumatoid OR gout) AND (thiazolidinedione OR thiazolidinediones OR TZD OR glitazone OR glitazones OR pioglitazone OR actos OR rosiglitazone OR avandia OR lobeglitazone OR duvie OR ciglitazone OR darglitazone OR englitazone OR netoglitazone OR rivoglitazone OR troglitazone OR rezulin OR balaglitazone OR drf-2593 OR as-605240)
Embase	(arthritis OR osteoarthritis OR polyarthritis OR rheumatism OR rheumatic OR rheumatoid OR gout) AND (thiazolidinedione OR thiazolidinediones OR TZD OR glitazone OR glitazones OR pioglitazone OR actos OR rosiglitazone OR avandia OR lobeglitazone OR duvie OR ciglitazone OR darglitazone OR englitazone OR netoglitazone OR rivoglitazone OR troglitazone OR rezulin OR balaglitazone OR drf-2593 OR as-605240) (Title) or (arthritis OR osteoarthritis OR polyarthritis OR rheumatism OR rheumatic OR rheumatoid OR gout) AND (thiazolidinedione OR thiazolidinediones OR TZD OR glitazone OR glitazones OR pioglitazone OR actos OR rosiglitazone OR avandia OR lobeglitazone OR duvie OR ciglitazone OR darglitazone OR englitazone OR netoglitazone OR rivoglitazone OR troglitazone OR rezulin OR balaglitazone OR drf-2593 OR as-605240) (Abstract)
Ovid	((arthritis or osteoarthritis or polyarthritis or rheumatism or rheumatic or rheumatoid or gout) and (thiazolidinedione or thiazolidinediones or TZD or glitazone or glitazones or pioglitazone or actos or rosiglitazone or avandia or lobeglitazone or duvie or ciglitazone or darglitazone or englitazone or netoglitazone or rivoglitazone or troglitazone or rezulin or balaglitazone or drf-2593 or as-605240)).mp. [mp = title, abstract, original title, name of substance word, subject heading word, floating sub-heading word, keyword heading word, organism supplementary concept word, protocol supplementary concept word, rare disease supplementary concept word, unique identifier, synonyms] {Including Related Terms}
ProQuest	ti((arthritis OR osteoarthritis OR polyarthritis OR rheumatism OR rheumatic OR rheumatoid OR gout) AND (thiazolidinedione OR thiazolidinediones OR TZD OR glitazone OR glitazones OR pioglitazone OR actos OR rosiglitazone OR avandia OR lobeglitazone OR duvie OR ciglitazone OR darglitazone OR englitazone OR netoglitazone OR rivoglitazone OR troglitazone OR rezulin OR balaglitazone OR drf-2593 OR as-605240))
PubMed	(arthritis OR osteoarthritis OR polyarthritis OR rheumatism OR rheumatic OR rheumatoid OR gout) AND (thiazolidinedione OR thiazolidinediones OR TZD OR glitazone OR glitazones OR pioglitazone OR actos OR rosiglitazone OR avandia OR lobeglitazone OR duvie OR ciglitazone OR darglitazone OR englitazone OR netoglitazone OR rivoglitazone OR troglitazone OR rezulin OR balaglitazone OR drf-2593 OR as-605240)
Scopus	TITLE-ABS ((arthritis OR osteoarthritis OR polyarthritis OR rheumatism OR rheumatic OR rheumatoid OR gout) AND (thiazolidinedione OR thiazolidinediones OR tzd OR glitazone OR glitazones OR pioglitazone OR actos OR rosiglitazone OR avandia OR lobeglitazone OR duvie OR ciglitazone OR darglitazone OR englitazone OR netoglitazone OR rivoglitazone OR troglitazone OR rezulin OR balaglitazone OR drf-2593 OR as-605240))
Virtual Health Library	(arthritis OR osteoarthritis OR polyarthritis OR rheumatism OR rheumatic OR rheumatoid OR gout) AND (thiazolidinedione OR thiazolidinediones OR TZD OR glitazone OR glitazones OR pioglitazone OR actos OR rosiglitazone OR avandia OR lobeglitazone OR duvie OR ciglitazone OR darglitazone OR englitazone OR netoglitazone OR rivoglitazone OR troglitazone OR rezulin OR balaglitazone OR drf-2593 OR as-605240)
Web of Science	(arthritis OR osteoarthritis OR polyarthritis OR rheumatism OR rheumatic OR rheumatoid OR gout) AND (thiazolidinedione OR thiazolidinediones OR TZD OR glitazone OR glitazones OR pioglitazone OR actos OR rosiglitazone OR avandia OR lobeglitazone OR duvie OR ciglitazone OR darglitazone OR englitazone OR netoglitazone OR rivoglitazone OR troglitazone OR rezulin OR balaglitazone OR drf-2593 OR as-605240) (Title) or (arthritis OR osteoarthritis OR polyarthritis OR rheumatism OR rheumatic OR rheumatoid OR gout) AND (thiazolidinedione OR thiazolidinediones OR TZD OR glitazone OR glitazones OR pioglitazone OR actos OR rosiglitazone OR avandia OR lobeglitazone OR duvie OR ciglitazone OR darglitazone OR englitazone OR netoglitazone OR rivoglitazone OR troglitazone OR rezulin OR balaglitazone OR drf-2593 OR as-605240) (Abstract)
Wiley Online Library	“(arthritis OR osteoarthritis OR polyarthritis OR rheumatism OR rheumatic OR rheumatoid OR gout) AND (thiazolidinedione OR thiazolidinediones OR TZD OR glitazone OR glitazones OR pioglitazone OR actos OR rosiglitazone OR avandia OR lobeglitazone OR duvie OR ciglitazone OR darglitazone OR englitazone OR netoglitazone OR rivoglitazone OR troglitazone OR rezulin OR balaglitazone OR drf-2593 OR as-605240)” in Abstract

## Appendix B. For Results

**Table A2:** The numbers of records identified by each search engine.

Search Engine	Number of Records
ACM Digital	122
BASE	98
EbscoHost	30
Embase	82
Ovid	164
ProQuest	11
PubMed	154
Scopus	114
Virtual Health Library	258
Web of Science	117
Wiley Online Library	32

**Table A3:** Reasons for exclusion at the eligibility stage.

Item	Exclusion Reason
Bongartz et al. Treatment of active Psoriatic Arthritis with the PPAR gamma-agonist pioglitazone: an open-label pilot study [105].	Duplicate record
Cuzzocrea et al. Rosiglitazone a ligands of the peroxisome proliferator-activated receptor-γ (PPAR-γ) reduce the evolution of murine type II collagen-induced arthritis [107].	Animal non-TMJs study
Xu et al. Implication for thiazolidinediones (TZDs) as novel potential anti-inflammatory drugs [108].	Review article
Stojanovska et al. The anti-atherogenic effects of thiazolidinediones [109].	Review article
Mahajan et al. Pioglitazone in experimentally-induced arthritis [110].	Animal non-TMJs study
Inokuchi et al. Effects of benzbromarone and allopurinol on adiponectin in vivo and in vitro [11].	Wrong drug
Fitzpatrick et al. The effects of rosiglitazone on bone by multiple image modalities in postmenopausal women with type 2 diabetes mellitus [111].	Conference abstract
Ormseth et al. Peroxisome proliferator-activated receptor gamma agonist treatment for rheumatoid arthritis: A proof-of-concept randomized controlled trial [112].	Conference abstract
Ormseth et al. Reversing vascular dysfunction in rheumatoid arthritis: improved augmentation index but not endothelial function with peroxisome proliferator-activated receptor γ agonist therapy [113].	No inflammation indicators
Kim et al. Changes in bone mineral density in patients with type 2 diabetes treated with lobeglitazone, a novel thiazolidinedione, over 52 weeks: A multicenter, randomized, double-blind, placebo controlled trial [114].	Conference abstract
Mohammed et al. Evaluation of the Clinical Use of Metformin or Pioglitazone in Combination with Meloxicam in Patients with Knee Osteoarthritis; using Knee Injury and Osteoarthritis Outcome Score [115].	No inflammation indicators
GlaxoSmithKline. A Randomised, Double-blind, Placebo-controlled, Parallel Group Study to Investigate the Anti-inflammatory and Metabolic Effects of Rosiglitazone XR, 8mg Once Daily, in Subjects With Rheumatoid Arthritis [116].	Results not publicly available
Chen et al. PPARγ is involved in the hyperglycemia-induced inflammatory responses and collagen degradation in human chondrocytes and diabetic mouse cartilages [117].	Cell line study
Zhu et al. PPARγ preservation via promoter demethylation alleviates osteoarthritis in mice [118].	Animal non-TMJs study
